# Encephalitis-Associated Human Metapneumovirus Pneumonia in Adult, Australia

**DOI:** 10.3201/eid2111.150608

**Published:** 2015-11

**Authors:** Anthony Fok, Cristina Mateevici, Belinda Lin, Ronil V. Chandra, Victor H.T. Chong

**Affiliations:** Author affiliations: Western Health, Melbourne, Victoria, Australia (A. Fok, C. Mateevici, B. Lin, V.H.T. Chong); Monash Health, Monash University, Melbourne (R.V. Chandra)

**Keywords:** encephalitis, human metapneumovirus, Paramyxoviridae, viruses, pneumonia, Australia

## Abstract

Human metapneumovirus pneumonia, most commonly found in children, was diagnosed in an adult with encephalitis. This case suggests that testing for human metapneumovirus RNA in nasopharyngeal aspirate and cerebrospinal fluid samples should be considered in adults with encephalitis who have a preceding respiratory infection,

Human metapneumovirus (HMPV) was first described in 2001 (*1*). HMPV is a member of the *Paramyxoviridae* family, the same family as respiratory syncytial, Nipah, Hendra, mumps, and measles viruses. HMPV has been reported worldwide and causes upper and lower respiratory tract infections, most commonly in children. A study in the Netherlands showed that, by 5 years of age, all children had been infected by HMPV (*1*). However, infection does not confer lifelong immunity; reinfection has been observed in adults and immunocompromised persons (*2*). During the past decade, HMPV respiratory infection associated with encephalitis has been documented in children (*3*–*7*). We present a case of encephalitis-associated HMPV infection in an adult in Australia.

## The Case

**Figure 1:**
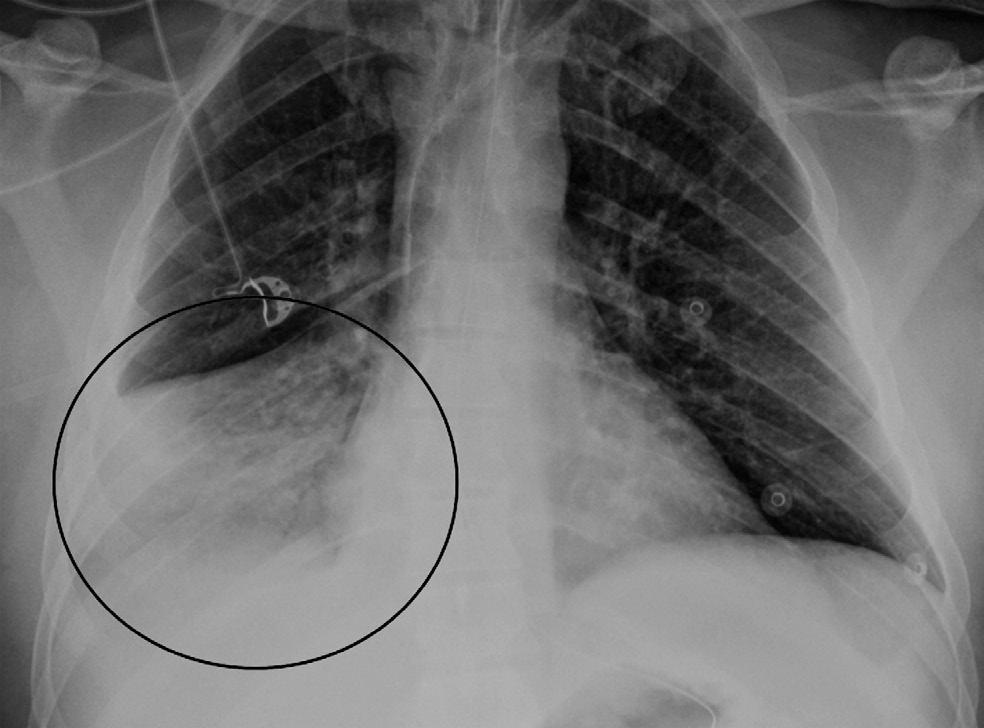
Frontal chest radiograph of a 47-year-old man with encephalitis-associated human metapneumovirus, Australia. Consolidation in the right middle lobe (circle) is compatible with pneumonia.

During the winter months of 2014 a 47-year-old man was found unconscious by his family at his home in Victoria, Australia. He had a 2-day history of upper respiratory tract symptoms (cough, dyspnea, rhinorrhea, myalgia, and headache). He had not traveled overseas recently. Emergency services personnel determined he had a Glasgow coma scale score of 10; in the emergency department, he was intubated when his Glasgow coma scale score dropped to 8. Examination showed blood pressure of 135/83 mm Hg, heart rate of 105 beats/min, and temperature of 37.2°C. He had no cranial nerve palsies; limb examination showed normal tone and reflexes; and he was moving all 4 limbs. He had mild neutrophilia (8.2 × 10^9^ cells/L [reference range 2.0–8.0]), mild lymphopenia (0.9 × 10^9^ cells/L [reference range 1.0–4.0]), and elevated C-reactive protein (30 mg/L [reference range <5]). Electrolytes, liver function, coagulation screen, thyroid function, ammonia, creatinine kinase, ethanol level, and paracetamol level were normal. Electrocardiogram demonstrated sinus tachycardia, and chest radiograph showed right basal pneumonia (Figure 1). At day 0, cerebrospinal fluid (CSF) showed glucose 4.2 mmol/L (reference range 2.0–3.9), protein 0.77 g/L (reference range 0.15–0.45), erythrocytes 43 × 10^6^ cells/L, and no leukocytes. Gram stain and culture were negative for microorganisms.

**Figure 2:**
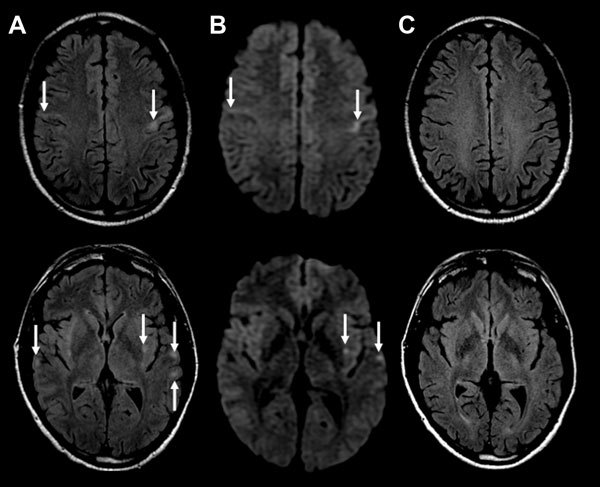
MRI findings from a 47-year-old man with encephalitis-associated human metapneumovirus pneumonia, Australia. A) Axial MRI FLAIR at presentation. Arrows indicate multiple areas of bilateral subcortical and external capsule FLAIR hyperintensities and perirolandic predominance (top image). B) Axial MRI DWI at presentation. Arrows indicate corresponding increase in DWI signal in the affected areas. C) Axial FLAIR MRI after 3 months. The MRI changes have all resolved. DWI, diffusion weighted imaging; FLAIR, fluid-attenuated inversion recovery; MRI, magnetic resonance imaging.

The patient was begun on acyclovir, ceftriaxone, vancomycin, and benzylpenicillin for suspected meningoencephalitis. CSF viral PCR was negative for *Mycoplasma pneumoniae*, herpes simplex viruses I and II, varicella zoster virus, and enterovirus. Tests for cytomegalovirus, *M. pneumoniae*, Epstein-Barr virus, and paraneoplastic antibodies and cytology were negative. Urine microscopy was unremarkable, and urine testing for *Legionella pneumophilia* serogroup 1 antigen, *Streptococcus pneumoniae* antigen, and *Chlamydia pneumoniae* antibody were negative. Repeat CSF examination on day 2 demonstrated glucose 4.2 mmol/L, protein 0.90 g/L, no erythrocytes, leukocytes 3 × 10^6^ cells/L, polymorphs 2 × 10^6^ cells/L, and mononuclear cells 1 × 10^6^/L. Gram stain and repeat viral testing results were unremarkable. Blood cultures did not grow microorganisms. Magnetic resonance imaging (MRI) with contrast showed bilateral subcortical and external capsule fluid-attenuated inversion recovery (FLAIR) and diffusion weighted imaging (DWI) hyperintensities, with perirolandic predominance (Figure 2, panels A, B) without leptomeningeal or parenchymal enhancement. Results of magnetic resonance angiogram were normal; there was no hemorrhage.

Given that CSF viral PCR was negative and no clinical improvement occurred, the patient was treated with a 5-day course of intravenous 1-g methylprednisolone for suspected autoimmune encephalitis/cerebral vasculitis. He was extubated on day 2 but had persistent confusion and agitation and was reintubated. He was subsequently extubated on day 4 when his confusion resolved. On day 4, clinical examination and electroencephalography were normal, and his nasopharyngeal aspirate (NPA) was positive for HMPV and negative for respiratory syncytial virus; influenza A and B viruses; parainfluenza viruses 1, 2, and 3; adenovirus; and picornaviruses. We had insufficient CSF for HMPV testing. Follow-up 3 months later indicated no residual deficits, and MRI demonstrated resolution of the acute changes (Figure 2, panel C).

## Conclusions

The clinical presentation, CSF results, and radiologic findings supported the diagnosis of encephalitis. CSF examinations showed elevated protein with no marked pleocytosis typically seen in viral encephalitis and similar to a case of respiratory HMPV with central nervous system (CNS) involvement in a child (*7*). Normal or near-normal CSF leukocyte counts have been reported in encephalitis-associated HMPV infection (*3*–*5*,*7*) and might reflect the lack of meningeal inflammation, which suggests that primary infection is through the respiratory tract and not through CNS invasion. The patient’s NPA PCR was negative for other viruses, except HMPV. Other viral encephalitides are less likely because common encephalitic viruses were not found on 2 occasions in the CSF. CSF samples were not blood stained and were unlikely to be falsely negative from heme products inhibition of PCR processes (*8*).

MRI findings were similar to those in 2 other encephalitis-associated HMPV cases (*6*,*7*). In a 10-year-old girl with encephalitis, HMPV was detected in NPA and CSF, and MRI showed cortical and subcortical T2 FLAIR hyperintensities with evolving DWI hyperintensities (*6*). Another case described cortical and subcortical FLAIR hyperintensities (*7*). In the case reported here, given the MRI DWI abnormalities, cerebral vasculitis and autoimmune encephalitis were differential diagnoses. However, the acute clinical presentation and benign course did not support either of these diagnoses. In addition, the resolution of DWI lesions and radiologic lack of disease activity at 3 months with 1 course of methylprednisolone rarely occurs in either of these diseases. MRI findings were not typical of herpes simplex virus–associated encephalitis. In Hendra virus–associated encephalitis, MRI shows similar microinfarcts with T2/FLAIR and DWI lesions (*9*). We did not test for Hendra virus because it has never been reported in Victoria and the patient had no contact with horses, the primary vector in Australia. The clinical course of Hendra encephalitis is longer and the outcome more severe. MRI findings in this patient suggested micro-infarcts involving the small vessels at the corticosubcortical junctions and deep white matter.

The limitation of this case was that we did not test for HMPV in CSF. HMPV encephalitis in adults has not been documented in the literature and at the time was not considered as a cause. We learned later in the patient’s admission about the positive HMPV NPA and did not have enough saved CSF for HMPV PCR testing. This limitation emphasizes an important teaching point: for all patients with encephalitis, a portion of CSF should be saved for future analyses and, if an etiology is not found in the initial CSF analysis, then less common but possible etiologies, such as HMPV, should be tested for with saved CSF. HMPV RNA was detected in NPA and CSF in 1 child with encephalitis (*6*). In other cases with NPA positive for HMPV, HMPV PCR has yielded negative results in CSF (*3*–*5*). In the case reported here, the negative test results for other infectious agents and positive NPA by HMPV PCR conclude that HMPV is the most likely causative agent for encephalitis. Asymptomatic carriage of HMPV is uncommon, and positive NPA by HMPV PCR in asymptomatic persons without any respiratory symptoms is rare (*1*,*10*).

Previously reported cases of HMPV with CNS involvement have occurred in children. Most of these reports describe respiratory symptoms before CNS involvement (*3*–*7*); in 1 case, HMPV RNA was found in brain and lung tissue on autopsy (*7*). This finding supports an etiologic agent with predilection for lung and brain infection but requires initial respiratory tract inoculation. The case reported here raises the possibility of HMPV causing encephalitis in adults with preceding respiratory infection. Testing for HMPV RNA in NPA and CSF should be considered not only in children with encephalitis but also in adults with encephalitis who have a preceding respiratory infection and CSF and radiologic abnormalities suggestive of a viral infectious agent.
